# Dynamics of Macrophage Trogocytosis of Rituximab-Coated B Cells

**DOI:** 10.1371/journal.pone.0014498

**Published:** 2011-01-17

**Authors:** Theodore Pham, Patricia Mero, James W. Booth

**Affiliations:** 1 Department of Immunology, University of Toronto, Toronto, Ontario, Canada; 2 Molecular and Cellular Biology Research, Sunnybrook Research Institute, Toronto, Ontario, Canada; Universidade de São Paulo, Brazil

## Abstract

Macrophages can remove antigen from the surface of antibody-coated cells by a process termed trogocytosis. Using live cell microscopy and flow cytometry, we investigated the dynamics of trogocytosis by RAW264.7 macrophages of Ramos B cells opsonized with the anti-CD20 monoclonal antibody rituximab. Spontaneous and reversible formation of uropods was observed on Ramos cells, and these showed a strong enrichment in rituximab binding. RAW-Ramos conjugate interfaces were highly enriched in rituximab, and transfer of rituximab to the RAW cells in submicron-sized puncta occurred shortly after cell contact. Membrane from the target cells was concomitantly transferred along with rituximab to a variable extent. We established a flow cytometry-based approach to follow the kinetics of transfer and internalization of rituximab. Disruption of actin polymerization nearly eliminated transfer, while blocking phosphatidylinositol 3-kinase activity only resulted in a delay in its acquisition. Inhibition of Src family kinase activity both slowed acquisition and reduced the extent of trogocytosis. The effects of inhibiting these kinases are likely due to their role in efficient formation of cell-cell conjugates. Selective pre-treatment of Ramos cells with phenylarsine oxide blocked uropod formation, reduced enrichment of rituximab at cell-cell interfaces, and reduced the efficiency of trogocytic transfer of rituximab. Our findings highlight that dynamic changes in target cell shape and surface distribution of antigen may significantly influence the progression and extent of trogocytosis. Understanding the mechanistic determinants of macrophage trogocytosis will be important for optimal design of antibody therapies.

## Introduction

Fcγ receptors (FcγR) expressed on phagocytic cells such as macrophages and neutrophils play an important role in mediating the clearance of IgG-opsonized foreign material from the body. FcγR can mediate several mechanistically distinct uptake processes. Large (>1 µm diameter) particles can be taken up by phagocytosis, where directed rearrangement of the actin cytoskeleton generates pseudopods that engulf the particle [1]. In contrast, small soluble immune complexes are internalized through clathrin-mediated endocytosis, where clathrin-coated pits pinch off from the plasma membrane to form endosomes. These two routes of internalization make use of different mechanisms and have different signalling requirements [2]–[4]. A distinct third situation is that in which the antibody is neither in a soluble complex nor bound to a rigid particle, but rather is bound to an antigen on the surface of a cell. In this case, though targeted antigen is confined to the target cell membrane, it is potentially mobile within the plane of that membrane, adding an additional level of complexity.

The term trogocytosis was coined to describe the phenomenon where surface molecules are extracted from one cell by another [5]. Many groups have demonstrated the ability of lymphocytes to trogocytose through their antigen receptors. T cells are able to acquire cognate MHC-peptide and associated membrane via their TCR [6]; this acquisition may be involved in the termination of the T cell response by conferring specificity to regulatory T cells [7], or by sensitizing antigen-specific T cells to fratricidal CTL-mediated killing [8]. B cells can internalize membrane-bound antigens via their B cell receptors, directing the antigens to MHC-loading compartments for subsequent presentation to T cells [9]. NK cells are able to acquire activatory ligands for NK receptors, thus becoming able to modulate the activity of other NK cells in *trans* [10]. These examples of trogocytosis occur at organized cell-cell interfaces, often termed immunological synapses.

Trogocytosis has also been studied in the context of FcγR-bearing macrophages removing surface antigen from IgG-opsonized cells. While this phenomenon was first observed over three decades ago in early studies of phagocytosis [11], it has only recently been studied in detail, in particular within the context of cells opsonized with the therapeutic anti-CD20 antibody rituximab (RTX), as well as several other therapeutic antibodies [12]–[14]. RTX is useful clinically because of its ability to promote the clearance of CD20-expressing B lymphoma cells. Taylor and coworkers have demonstrated that both RTX and CD20 are removed from RTX-opsonized B cells by trogocytosis *in vitro* [12] and *in vivo* [15]. This is of substantial clinical significance, as the removal of CD20 limits the therapeutic efficacy of RTX by impairing the clearance of tumor cells that normally occurs via mechanisms of antibody-dependent cellular cytotoxicity, complement-dependent cytotoxicity, phagocytosis, or the induction of apoptosis [16]. Given the rapidly increasing role of therapeutic antibodies in medicine [17], a greater understanding of the factors that influence macrophage trogocytosis will be important for improved design of such therapies.

While FcγR-mediated trogocytosis has been previously demonstrated by several groups, the dynamics of this process – how macrophages and target cells interact in real time - has been little explored. We sought to better understand these dynamics by following the process using live cell microscopy. For FcγR-bearing cells we used RAW264.7 cells, a mouse macrophage cell line that has been used extensively for studying FcγR-mediated phagocytosis. As the trogocytic target, we used cells of the Ramos B lymphoma cell line, opsonized with RTX. We hypothesize that target cells may not simply be passive players in trogocytosis. Indeed, we find that trogocytosis is a highly dynamic process in which rearrangements of ligand on the opsonized target cell can play an active role.

## Results

### Dynamic morphology of Ramos cells and trogocyte capture of RTX-rich uropodia

**Figure 1:**
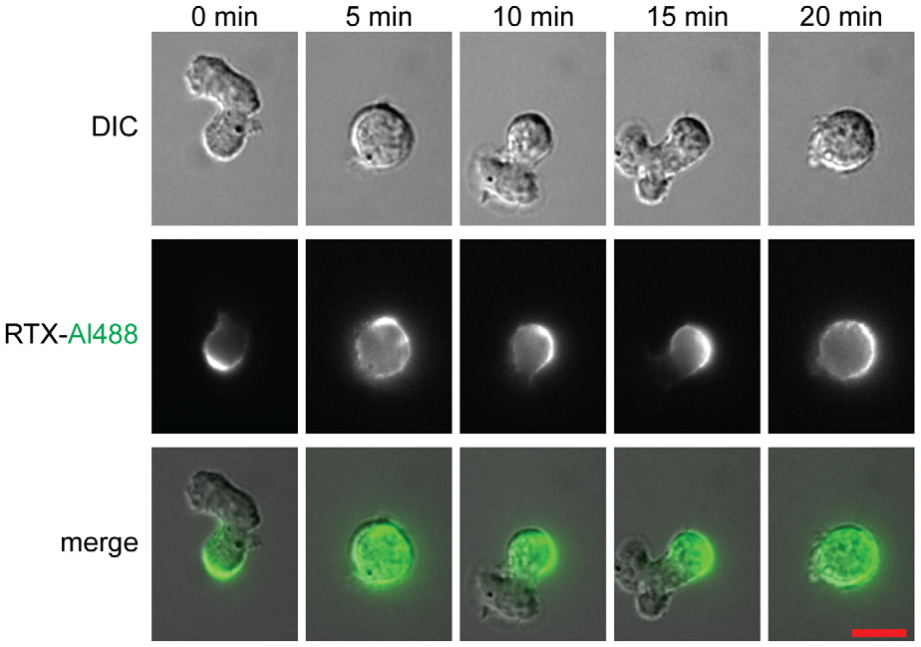
Dynamic morphology of Ramos B cells. Selected frames from live microscopic imaging of RTX-Al488 coated Ramos cells (Videos S1). Ramos cells were able to adhere to the substratum by the formation of a uropod as seen at the first time of observation (0 min), and at 10 and 15 minutes afterwards. RTX-Al488 (green) was enriched at uropods, while being depleted from the opposite, mobile end. Scale bar 10 µm.

We sought to follow the progression of trogocytosis by examining the interaction of opsonized Ramos cells with adherent RAW cells by time lapse fluorescence microscopy. Notably, it was immediately apparent that even in the absence of interaction with a phagocytic (or “trogocytic”) cell, the distribution of Alexa Fluor 488-labeled RTX (RTX-Al488) on the opsonized Ramos cells was not uniform. The Ramos cells were motile, and alternated between displaying a round morphology and fluctuating morphology with extensive lamellipodia (Figure 1, top row). This behaviour was independent of RTX opsonization (data not shown). While RTX was homogenously distributed on the surface of round cells, on cells with lamellipodia the RTX-Al488 signal became strikingly polarized to the end of the cell opposite the lamellipodial extensions (Figure 1, middle row). In most cases cells “squirmed” in one location with a CD20-positive uropod adherent to the substratum, while in others, Ramos cells moved over extended distances, with RTX concentrating at the back end of the cell (Videos S1 and S2). Polarized localization of CD20/RTX was robust; at any given moment in time, 12±3% of Ramos cells displayed a polarized morphology, and 84±1% of these cells showed polarization of RTX to the end of the cell opposite lamellipodial extensions (± s.d., n = 4 experiments, with >300 cells scored/experiment). This polarization of RTX was rapidly reversible; a return to the round morphology was accompanied by the homogenous redistribution of RTX.

**Figure 2:**
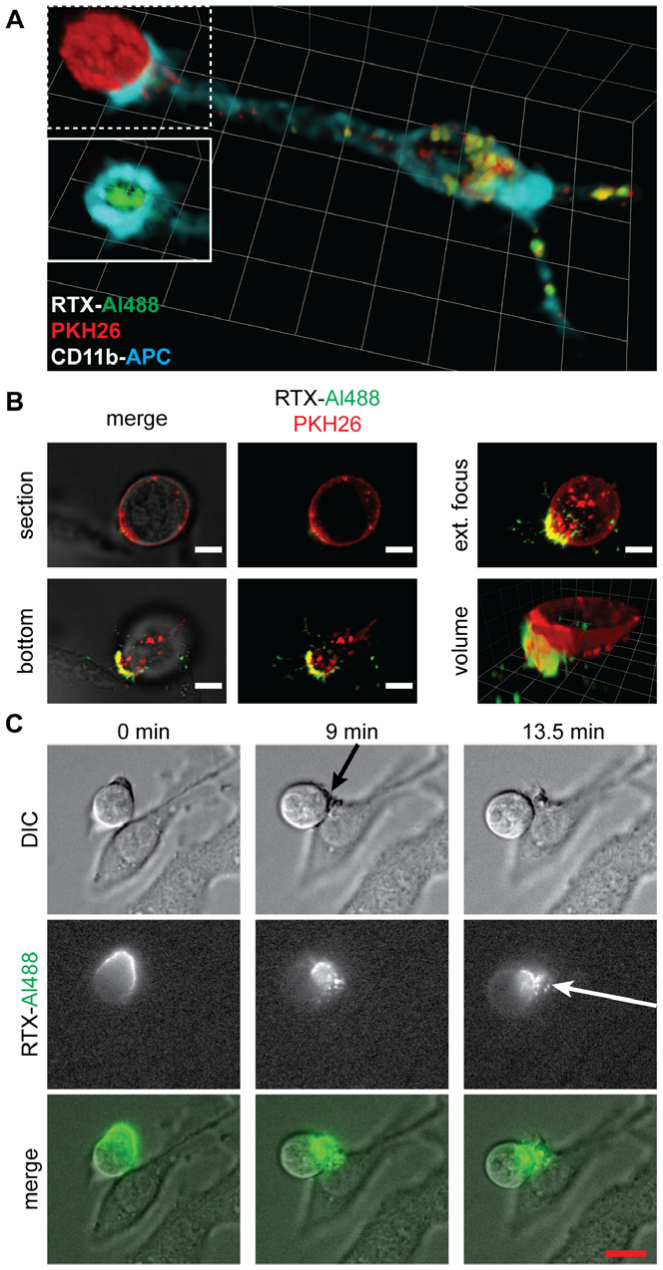
Concentration of RTX at RAW-Ramos interfaces. (A) Volumetric reconstruction from confocal slices of a Ramos-RAW cell interface. RTX-Al488-coated (green), PKH26-labelled Ramos cells (red) were incubated with RAW cells for 45 minutes at 37°C. RAW cells were labelled with anti-CD11b-APC (cyan). The RAW cell has extensively trogocytosed both RTX and PKH26. Inset shows the dotted area above it without the PKH26 channel overlaid, revealing the concentration of RTX-Al488 at the cell-cell interface, otherwise depleted from the rest of the Ramos cell. Trogocytosis reaction was halted by fixation 45 min after co-incubation. Ramos cells are approximately 12 µm in diameter. (B) Deconvolved epifluorescence images from live experiment of PKH26-labelled, RTX-Al488 coated Ramos after coincubation with RAW cells for 1 hour. Extended focus and volumetric representations of the imaged slices show accumulation of RTX-Al488 at the interface. Scale bar 5 µm. Unit size for volume 3.3 µm. (C) Frames from live microscopic imaging following a RTX-Al488 coated Ramos cell settling onto a RAW macrophage. Extensive RAW membrane ruffling occurs to capture the uropod (black arrow, 9 minutes), and RTX is internalized shortly after (white arrow, 13.5 minutes). See Videos S3. Scale bar 10 µm.

**Figure 3:**
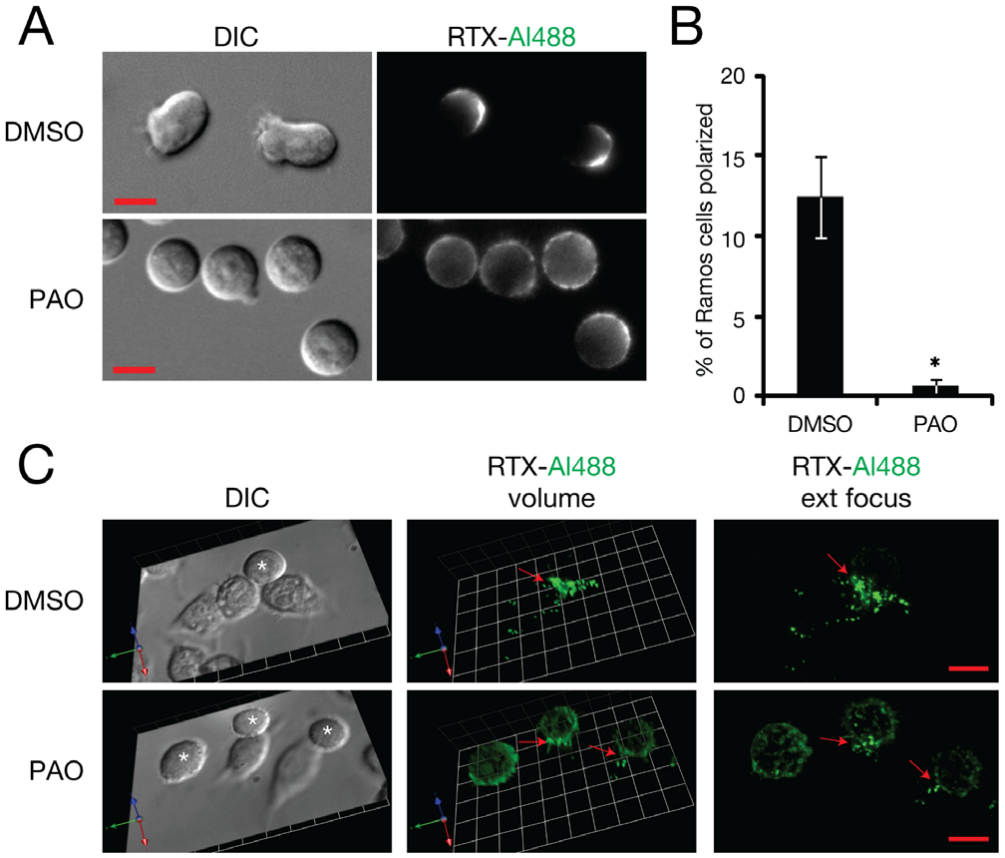
Effect of treatment of Ramos cells with PAO on cell polarization and RTX accumulation at cell-cell interfaces. Opsonized Ramos cells were treated with 30 µM PAO or DMSO vehicle for 10 min then washed. (A) Cell morphology and RTX distribution on live Ramos cells. (B) Quantitation of effect of PAO on Ramos cell polarization. Images of live cells were acquired after treatment with PAO or DMSO, and the fraction of total cells showing a polarized morphology at the moment of image acquisition was scored. Error bars show standard deviation (n = 4 experiments, with >300 cells scored per condition in each experiment (p<0.05)). (C) Comparison of RTX accumulation at the interfaces between RAW and Ramos cells with or without pretreatment of Ramos cells with PAO. Deconvolved volumes and extended focus representations of target-trogocyte interfaces are presented. Interfaces were imaged after 15 minutes of allowing cells to interact. Arrows indicate zones of contact between RAW and Ramos (*) cells. Similar results were seen in 3 independent experiments. Scale bars 10 µm.

This non-uniform distribution of CD20 clearly has important implications for the interaction of opsonized Ramos with RAW cells. Of note, RAW cells formed conjugates with Ramos in which striking concentration of RTX was seen at the cell-cell interface (Figures 2A and 2B). Prior polarization of CD20 may favour trogocytosis by concentrating antigen in one location. Indeed, on occasion, RAW cells appeared to capture polarized Ramos by binding to the RTX-positive end (Figure 2C, Videos S3 and S4). RAW cells were able to interact with multiple Ramos cells at once; Video S5 shows a RAW cell with several bound motile Ramos, in each of which RTX-Al488 is concentrated at the cell-cell interface. Capture of Ramos via a pre-existing RTX-enriched polarized end provides one possible explanation for the observed concentration of RTX at interfaces; it is also possible, however, that Ramos polarization occurs concurrently with or subsequent to binding to the RAW cells. Alternatively, forces exerted by the RAW cells via their FcR may drive the local clustering of RTX. These possibilities are not mutually exclusive; both cells may contribute in a concerted fashion to forming a concentrated zone of RTX. Since the changes in Ramos morphology are rapid and reversible, simply observing capture events does not allow one to distinguish the respective roles of trogocyte and target cell in forming this enriched contact zone. To address the question of what effect Ramos-driven polarization of CD20 has on the trogocytic interaction, we sought to prevent this polarization by pharmacological inhibition. We first attempted to use cholesterol extraction of the Ramos cells with methyl-β-cyclodextrin to disrupt membrane microdomains,which may contribute to the subcellular localization of RTX [18]. Moderate extraction conditions (10 mM methyl-β-cyclodextrin for 10 min) did not abolish uropod formation. More prolonged treatment (30 min) did have an inhibitory effect; however, it also resulted in a ten-fold decrease in the extent of RTX opsonisation, suggesting that extensive cholesterol depletion prevents efficient RTX binding to CD20 (data not shown). As an alternative approach, we tried pretreating Ramos cells with the phosphatase inhibitor phenylarsine oxide (PAO) to promote a general disruption of signalling pathways. We found that treatment with PAO led to a loss of cell motility and uropod formation that was irreversible over the time scale of our experiments (Figure 3A, B). This allowed us to treat Ramos cells with PAO, then wash away the PAO and examine the interaction of the pretreated Ramos with RAW cells. We observed that concentration of RTX at cell-cell interfaces was substantially reduced by pre-treatment of Ramos with PAO (Figure 3C). This suggests that polarization of the target cell contributes to the localized accumulation of RTX at cell-cell interfaces.

### Internalization of RTX-Al488 by RAW cells

**Figure 4:**
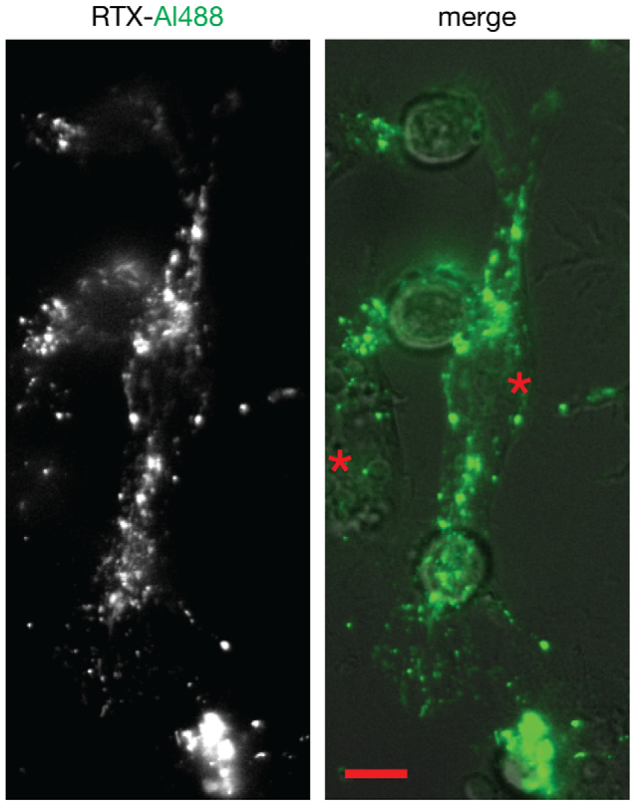
Distribution of trogocytosed RTX within RAW cells. Acquired RTX-Al488 becomes widely distributed in RAW cells (*). Image taken 70 minutes after the addition of RTX-Al488 coated Ramos. Scale bar 10 µm. See Video S6.

Within minutes of capture, small sub-micron RTX-Al488 positive puncta became visible moving inside the RAW cells near the site of contact (Videos S3 and S4). Over longer times, these became widely distributed throughout the RAW cells (Figure 4), where they appeared to stream in a directed manner (Video S6). At late times, RTX-Al488 was found in large vacuolar compartments in many cells (Video S7).

### RTX-dependent transfer of target cell membrane to trogocyte

**Figure 5:**
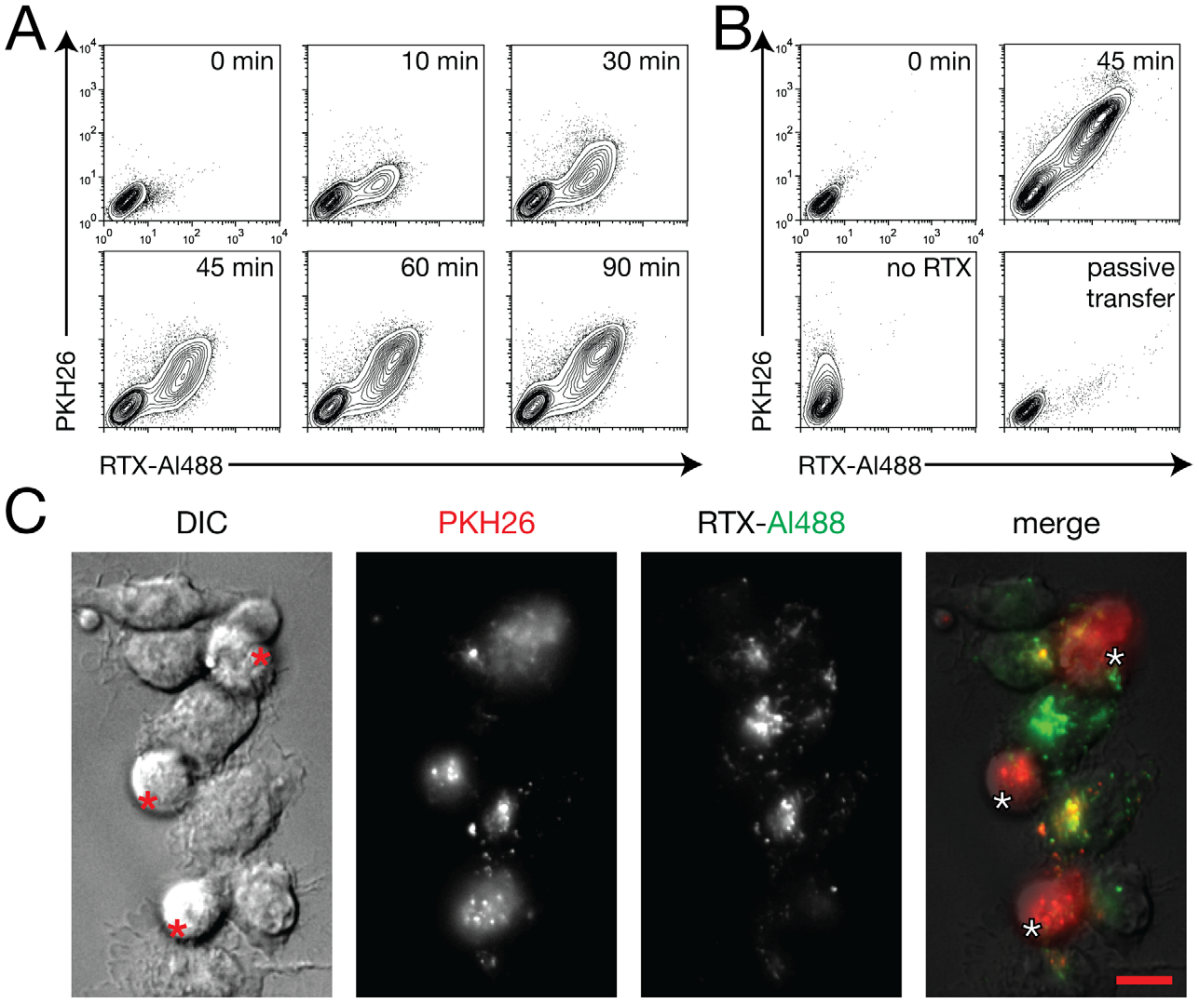
Cotransfer of PKH26 and RTX. (A) Simultaneous acquisition of PKH26 and RTX-Al488 by RAW cells from Ramos cells was analyzed by flow cytometry. (B) Lack of acquisition of PKH26 and RTX-Al488 by RAW cells after 45 minutes when opsonization of Ramos cells was omitted (no RTX), or when RAW cells were provided with culture supernatants of RTX/PKH labelled Ramos cells incubated for the same amount of time (passive transfer). (C) RAW cells that have internalized mainly RTX (green), or both RTX and PKH26 (yellow). Ramos cells indicated by asterisks. Scale bar 10 µm.

It has been demonstrated in previous studies of trogocytosis using RTX-opsonized targets that not only antibody, but also CD20 and accompanying fragments of target cell membrane are transferred to the trogocyte [12], [13]. To confirm similar transfer in our system, we labelled Ramos cells with the membrane dye PKH26 before opsonization. By flow cytometry, we observed that PKH26 is indeed transferred from the Ramos to RAW cells (Figure 5A). Interestingly, PKH26 acquisition was not directly proportional to RTX transfer throughout the trogocytic reaction (Figure 5A). Early during trogocytosis, it appears that more RTX is transferred per amount of bulk membrane. The enrichment of RTX at interfaces may initially favour either transfer of RTX/CD20 complexes with less accompanying membrane, or enhanced transfer of RTX alone. Observation of RTX-Al488 positive compartments in RAW cells after trogocytosis by microscopy similarly revealed heterogeneity in the extent of co-transfer of PKH26; in some cells, there was significant PKH26 label in all compartments that had RTX, whereas in others, relatively little concomitant PKH26 transfer was seen (Figure 5C). Transfer of PKH26 was dependent on RTX opsonization of Ramos cells (Figure 5B); also, both RTX and PKH26 were acquired directly from the target cell rather than through passive transfer by release into the medium, as no transfer of either label was seen when RAWs were incubated for 45 min with culture supernatants from RTX/PKH26 labelled Ramos cells that had been incubated for 45 min to allow for possible release of label (Figure 5B).

### Kinetics of acquisition and internalization of RTX by RAW cells

**Figure 6:**
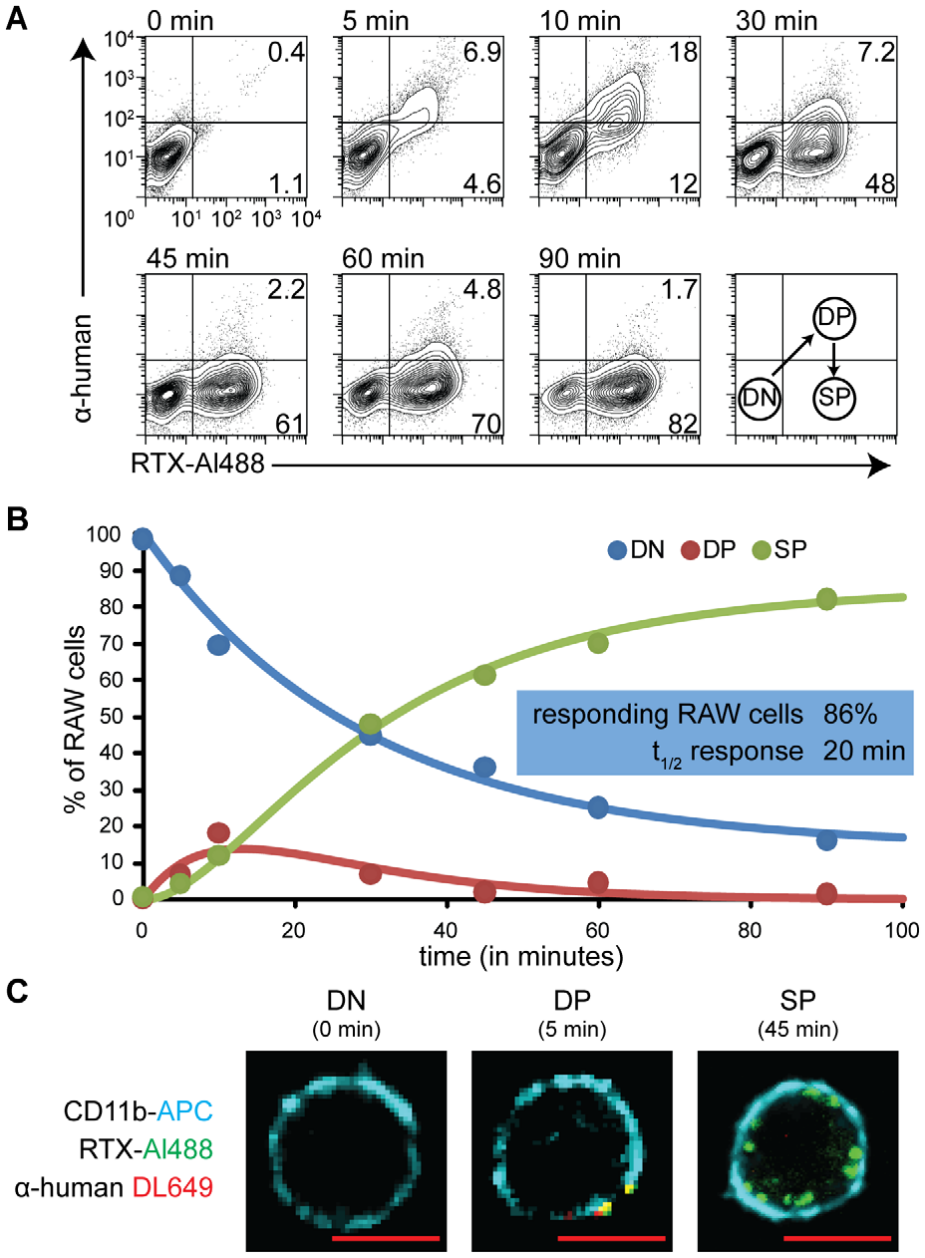
Kinetic analysis of acquisition and internalization of RTX by flow cytometry. Internalization of acquired RTX was assessed by its inaccessibility to labelling with anti-human antibody. (A) RTX-Al488 coated Ramos cells were coincubated with RAW cells at 37°C for the times indicated. After staining with anti-human antibody, RAW cells were detached and identified by gating on CD11b+ events. Bottom right plot shows a schematic of the progression of RAW cells in the kinetic analysis of trogocytosis. Indicated numbers are percentage of events in each quadrant out of the DN, DP, and SP populations. (B) The proportion of RAW cells in the DN population was fitted to a model as described in Methods. (C) Confocal slices of cells representative of trogocytic stages. Scale bar 10 µm.

It is clear from microscopy that the RTX is not only transferred to the RAW cell but becomes internalized, as was also seen in earlier studies of macrophage trogocytosis [12]. We wanted to analyze this process by flow cytometry and differentiate between internalized and surface-exposed RTX over time. To this end, we incorporated labelling of surface-exposed RTX on RAW cells with anti-human antibodies (recognizing the Fc portion of this chimeric antibody). After incubation together, RAW cells were washed, detached, and then distinguished from remaining Ramos cells by staining for CD11b. Cells were then incubated with anti-human antibodies. Using this analysis, three populations of RAW cells were observed to develop over time. Initially, the RAW cells were negative for both RTX and anti-human antibody (Figure 6A; DN quadrant). Then over time a population arose that was positive for both RTX and anti-human (Figure 6A, DP quadrant). This represents trogocytic intermediates in which transfer but not internalization of RTX has occurred; these may either be free RAW cells that have detached a piece(s) of Ramos cells, or the RAW cell halves of RAW-Ramos conjugates that are dissociated in the process of detachment and resuspension of the RAW cells (few if any conjugates remained intact after RAW detachment (Figure S1A)). In either case, appearance of this population represents a first step in which RTX becomes associated with the RAW cell prior to its internalization. Finally, double-positive cells disappeared and RTX single-positive cells appeared (Figure 6A, SP quadrant), as RTX became internalized into the RAW cell and was no longer accessible to the added anti-human antibody. Thus, this analysis highlights that trogocytosis by macrophages can be thought of as involving two sequential steps: conjugate formation and antigen internalization.

### Effects of chemical inhibitors on kinetics of trogocytosis

**Figure 7:**
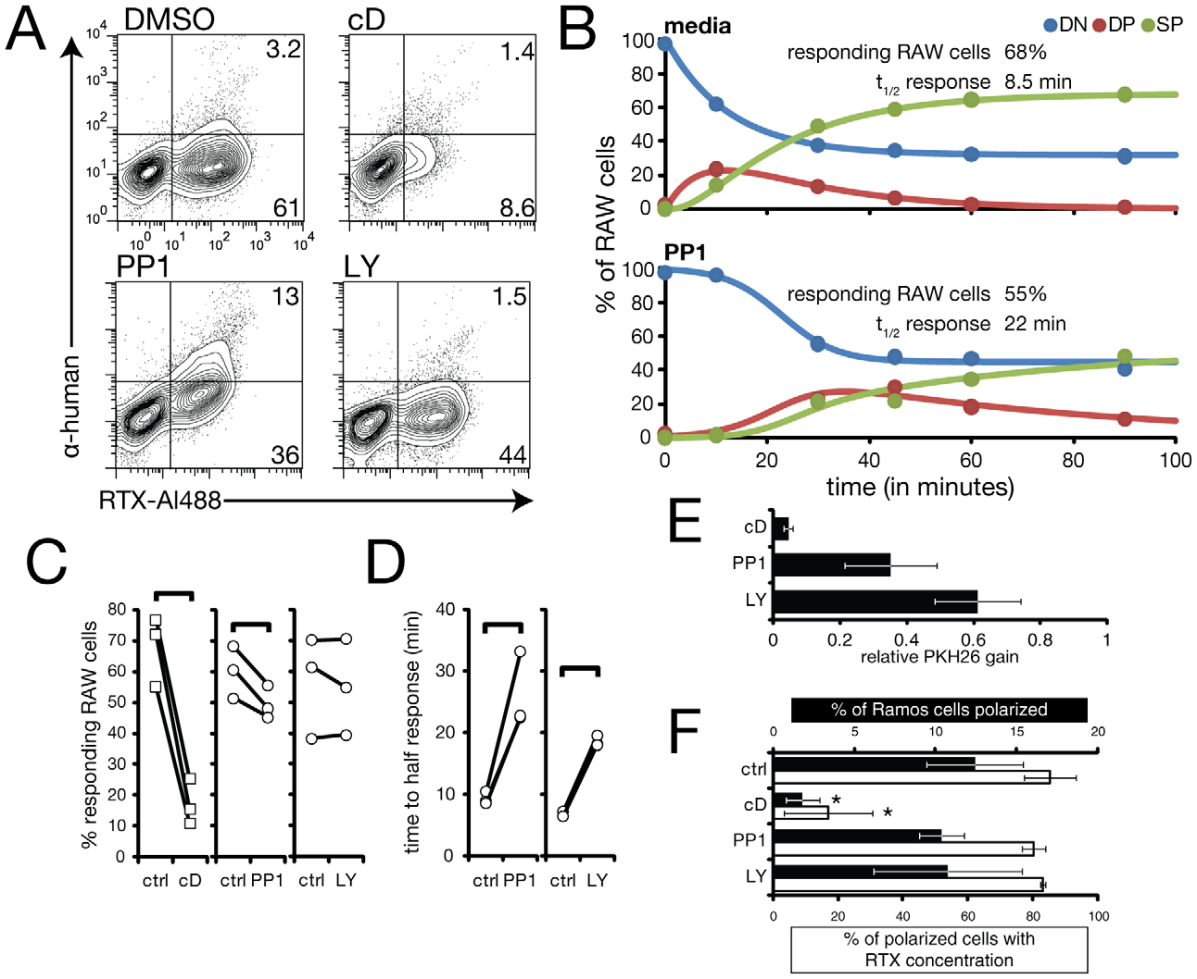
Kinetic analysis of chemical inhibition of trogocytosis. (A) Analysis of RTX transfer and internalization as in Figure 5 after 45 min of trogocytosis in the presence of indicated inhibitors. cD, cytochalasin D. (B) Example kinetics of control versus PP1-inhibited trogocytosis. (C) Comparison of percent responding RAW cells under different conditions. Linked pairs indicate individual experiments. Braces indicate significance (p<0.05), n = 3 for each control-inhibitor pair. For cytochalasin D experiments, percent responding RAW cells (control and cytochalasin D-treated) are indicated by the percent responding at 90 minutes rather than the modelled asymptote. (D) Comparison of time to half-response under different conditions. (E) Effect of inhibitors on acquisition of PKH26 at 90 minutes relative to media-treated RAW cells. Error bars indicate standard deviation, n = 3 for each treatment. (F) Effect of inhibitors on Ramos cell polarization (black bars) and concentration of RTX at uropods (white bars). Error bars indicate standard deviation. Only cytochalasin D causes significant inhibition (p<0.05, n = 4).

FcR-mediated phagocytosis requires extensive rearrangement of the actin cytoskeleton triggered by Src family kinase-mediated signalling downstream of receptor engagement [19]. Phosphatidylinositol 3 kinase (PI3K) is also required for engulfment of large particles in a size-dependent manner, and acts to synthesize PIP_3_ at early stages of phagocytosis [20], [21]. Both Src family-kinase mediated protein phosphorylation and PIP_3_ levels are low in unstimulated RAW264.7 cells [20], [22]. To investigate the role of these players in trogocytosis, we tested the effect of several inhibitors, namely, cytochalasin D, an actin depolymerizing agent; PP1, a Src family kinase inhibitor; and LY294002, a PI3K inhibitor, at concentrations known to efficiently inhibit phosphorylation or phagocytosis in RAW264.7 cells [4], [21], [22] (Figure 7). For these experiments we pre-labelled Ramos cells with PKH26 and used a gating strategy in which Ramos cells remaining after detachment of adherent cells were identified based on their high staining for both PKH26 and anti-human (Figure S1D); fortuitously, Ramos cells are strongly positive for anti-human antibody staining even in the absence of RTX opsonization, presumably due to recognition of surface Ig. We assessed the effect of inhibitors on the fraction of RAW cells that moved out of the DN population and on the kinetics of progression from DN to DP to SP population. Cytochalasin D had the most profound effect, largely blocking acquisition of RTX (Figure 7C), as was also reported in previous studies of trogocytosis [12]. Though a small proportion of cytochalasin D-treated RAW cells moved into the SP population, these events failed to be PKH26 positive (Figures 7E and S2), suggesting they represent either internalization of only the most highly clustered RTX/CD20 complexes, or alternatively internalization of RTX alone. PP1 also resulted in a lower fraction of RAW cells responding, while LY294002 had little if any effect on the fraction of responders (Figure 7C). Both PP1 and LY294002 led to a delay in acquisition of RTX, with an increase in the half-time of movement from the DN to DP population, suggestive of a defect in conjugate formation in the presence of either inhibitor (Figure 7D). The inhibitors also all led to reduced transfer of PKH26 to the RAW cells (Figure 7E). Despite the delay in RTX acquisition, the cells still did eventually progress to the SP population, suggesting that while Src family kinases and PI3K are both required for optimal conjugate formation, neither is essential for RTX internalization. Treatment of Ramos cells on their own with PP1 or LY294002 did not affect either cell polarization or RTX concentration at uropods, suggesting that the effect of these inhibitors on trogocytosis was due to their effects on the RAW cell, not on the targets (Figure 7F). Cytochalasin D treatment did cause a large reduction in uropod formation (Figure 7F), which may contribute to the inhibition of trogocytosis (see below), though it is likely that the near-complete inhibition of trogocytosis by cytochalasin D is primarily due to its effect on the RAW cells.

### Effects of blockade of uropod formation on trogocytosis

**Figure 8:**
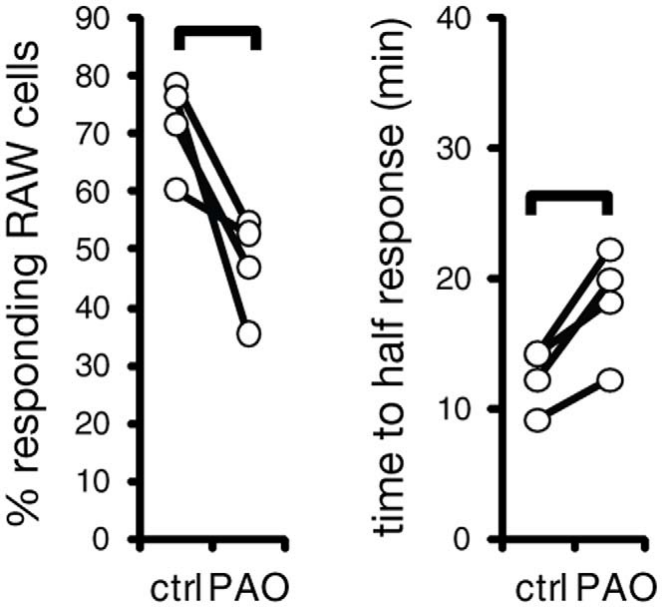
Effect of pre-treatment of Ramos cells with PAO on trogocytosis. Ramos cells were pretreated with 30 µM PAO for 10 min and washed before addition to RAW cells. Trogocytosis of RTX was analyzed by flow cytometry for percent responding RAW cells and time to half-response. Linked pairs indicate individual experiments. Braces indicate significant difference (p<0.05, n = 4).

To assess the effect of polarization of target cells on the efficiency of trogocytosis, we pre-treated Ramos cells with PAO (followed by washing away PAO) to prevent formation of RTX-rich uropods, as described above. As noted above, this treatment led to reduced RTX concentration at sites of cell-cell contact (Figure 3C). It also led to both a significant reduction in the fraction of RAWs that are able to successfully engage Ramos and a delay in the kinetics of RTX transfer (Figure 8). Taken together, these results suggest that polarized concentration of antigen on a target cell can facilitate the process of trogocytosis of that antigen.

## Discussion

We observed a striking accumulation of CD20 at the uropod of motile Ramos cells. CD20 has been shown to reside in “raft” microdomains by biochemical fractionation [23], or RTX colocalization with cholera toxin subunit B staining [24], so it is possible that its polarization is related to an association with particular membrane subdomains. The polarization may be a consequence of RTX binding/clustering, or alternatively may occur even in the absence of RTX. These observations make the important point that the mobility and distribution of a targeted antigen in the cell membrane will likely be a key parameter in determining the extent of trogocytosis. Polarized localization of antigen may favour trogocytosis while conversely impairing or even preventing complete phagocytosis of cells. FcR-mediated phagocytosis proceeds by a “zippering” mechanism, in which sequential engagement of Fc receptors by opsonizing antibody drives pseudopods to extend around the particle. The absence of opsonin on one side of a target prevents progression of pseudopod extension [11], [25]. In fact, the inability of macrophages to perform phagocytosis of capped lymphocytes (where antibody-bound surface Ig was polarized to one end of the lymphocyte) was a key finding that provided an elegant and unambiguous proof of the zipper hypothesis for phagocytosis [11]. In a similar manner, the polarized localization of RTX/CD20 at the cell-cell interface in our system may halt the circumferential extension of pseudopods around the Ramos cell and, conversely, facilitate the transfer of CD20/RTX complexes by trogocytosis, as suggested by our findings of inhibition of trogocytosis when Ramos cells were pretreated with PAO to prevent polarization. While trogocytosis and phagocytosis can occur simultaneously under some conditions [14], we never observed complete phagocytosis of Ramos by RAW cells; this may be a consequence of CD20/RTX localization, or alternatively may simply be a consequence of the relatively large size of the Ramos – approximately 12 µm in diameter. Particle shape is a key parameter constraining the efficiency of phagocytosis [26], [27]. Only certain geometries of the interface between phagocyte and particle allow engulfment, which requires complex localized changes in phosphoinositides and signalling proteins at the phagocytic cup to orchestrate coordinated rearrangements of the cytoskeleton [19]. When the “particle” engaged by a macrophage is not inert (such a polystyrene bead) but rather a dynamic cell that can change both its overall shape and the surface distribution of opsonizing antibodies, clearly the phagocyte-target interaction involves an even greater complexity. Accordingly, to understand trogocytosis and phagocytosis of opsonized cells and the relative extent of these two processes, the characteristics of not only the trogocyte but also of both the target antigen and cell need to be considered. Recent work has demonstrated that certain plasma membrane proteins are preferentially transferred to lymphocytes by trogocytosis [28]. Similarly, the extent of antibody-dependent trogocytosis of antigen by monocytes varies for different antigens [13]. An important goal for future studies will be defining the features of targeted antigens (*e.g.* mobility in the plasma membrane) that influence trogocytosis. It will also be useful to incorporate modulation of structural features of the target cell such as membrane fluidity in these studies.

Our results with inhibitors implicate a role for cytoskeletal rearrangements, Src family kinases, and PI3K signalling in the optimal formation of cell-cell conjugates required for efficient trogocytosis. In contrast to our results, Beum *et al.* did not observe any effect of PP2 or wortmannin (also inhibitors of Src family kinases and PI3K, respectively), on the overall efficiency of transfer of RTX to THP-1 monocytes by trogocytosis [12]. One relevant difference between the two sets of findings may be the particular experimental configurations: in the THP-1 experiments, cells were brought together by centrifugation, whereas in our setup, the target cells come into contact with adherent RAW cells simply by landing on or near them under the influence of gravity. In the latter situation, the requirements for active pseudopod extension by the RAW cells in order to form a robust conjugate may be more stringent. This emphasizes that the trogocytosis process measured *in vitro* will be highly dependent on the particular experimental design. It is unclear what method of establishing cell-cell contact best reflects the *in vivo* scenario, where trogocytosis is likely to depend strongly on physical features of the local physiological microenvironment such as shear stresses.

**Figure 9:**
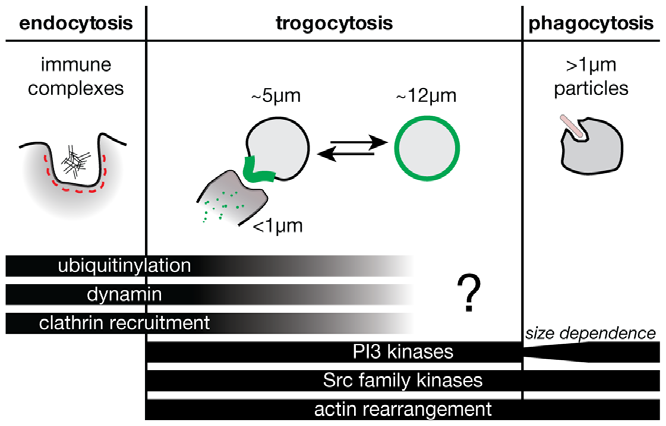
Trogocytosis may involve components of both the phagocytosis and endocytosis machinery.

An advantage of our method of analysis by flow cytometry is that it parses trogocytosis into two sequential steps: conjugate formation and antigen internalization by the trogocyte. The initial formation of cell-cell conjugates seems likely to involve many of the same cytoskeletal and membrane rearrangements required for phagocytosis of large particles, as is suggested by the effects of inhibitors we observe (Figure 9). The subsequent internalization of RTX from the target cells may also involve actin-driven force generation. Alternatively, mobile antigens that can be concentrated into small clusters on the target cell membrane may allow for clathrin-mediated uptake processes similar to those involved in endocytosis of soluble immune complexes. Our observation of trogocytosis in real time reveals that RTX is transferred in small puncta rather than large fragments. Moreover, the fact that PP1 and LY294002, inhibitors of phagocytosis, do not prevent internalization is consistent with an endocytic internalization mechanism. Our preliminary findings also suggest that transfection of RAW cells with a dominant negative form of dynamin, a GTPase required for membrane scission during FcγR-mediated endocytosis [4], inhibits trogocytic internalization of RTX (data not shown). It would be interesting in the future to examine the effect of specific perturbations of the endocytosis machinery, *e.g.* knockdown of clathrin-coated pit components. It may be that multiple internalization pathways are in fact initiated simultaneously, and which one predominates depends on several factors such as opsonin density and the fluctuating geometry of the particular cell-cell conjugate. More broadly, it is likely that the transfer of membrane-bound antigens between cells can be mediated by a variety of mechanisms; for instance, it has been shown that B cells are able to acquire membrane-bound antigens at 4°C, in contrast to T cells [18]. The requirement for conjugate formation to occur prior to antigen internalization poses a challenge for analyzing this second step of internalization in isolation.

While trogocytosis by T cells can lead to prolonged exposure of the trogocytosed antigen on the T cell surface [7], [29], in the case of macrophages, internalization of the removed antigen is likely the rule [30]. Thus, the functional implications of FcR-driven trogocytosis may relate not so much to macrophages gaining novel surface proteins (and hence novel functions) as has been seen with T and NK cells, but rather to its effects on the target cell. Removal of CD20 from B cells limits the therapeutic efficiency of RTX [31]. Conversely, for other antibodies the opposite may conceivably be the case. For example, one could speculate that removal of the growth-promoting Her2 receptor from breast cancer cells via macrophage trogocytosis of trastuzumab/Her2 complexes might actually help to reduce tumour cell survival, thereby enhancing the efficacy of trastuzumab. An additional important consequence of trogocytosis is the concomitant downregulation of FcγR on the trogocytic cell [13]. This downregulation may in fact account for the efficacy of RTX in treatment of some autoimmune diseases, by inhibiting FcγR-driven pathological inflammation (the “immune complex decoy hypothesis” [32]). Finally, trogocytosis may be a significant route of uptake of antigen leading to ultimate presentation to T cells [33]. For all these reasons, it will be important to determine both the mechanisms within the trogocytosing cell that are involved in antigen capture, as well as how the particular physical properties of the antigen and target cell affect the efficiency of trogocytosis.

## Materials and Methods

### Cell lines and culture conditions

RAW264.7 (RAW) cells were obtained from the American Type Culture Collection and maintained in DMEM with 10% heat-inactivated fetal bovine serum (FBS) (Wisent, Quebec, Canada). Cells were detached for subculturing or analysis by scraping. Ramos cells, a human Burkitt's B cell line, were generously provided by Dr. Alberto Martin (University of Toronto) and were maintained in IMDM (Gibco) + 10% FBS. All cell lines were grown in a humidified 37°C, 5% CO_2_ incubator.

### Reagents

Rituximab (RTX) was a generous gift from Dr. David Spaner (Sunnybrook Research Institute, Ontario, Canada) and was conjugated using an Alexa Fluor 488 Protein Labelling Kit (Invitrogen). Rat anti-mouse CD11b-PE/APC (clone M1/80) was obtained from the Sunnybrook Research Institute antibody facility, BD Pharmingen, or Biolegend and used as directed. Donkey anti-human DyLight 649 (DL649) antibody was obtained from Jackson ImmunoResearch. PP1 was from Biomol. LY294002 was from Calbiochem. Cytochalasin D, PKH26, dimethylsulfoxide (DMSO) and PAO were from Sigma.

### Preparation of cells for trogocytosis

Ramos cells were opsonized with Alexa Fluor-488-conjugated RTX (RTX-Al488) for 20 minutes with agitation at 37°C. RTX-Al488 was used at a saturating concentration of 0.3 µg per 10^6^ Ramos cells. In some experiments, Ramos cells were labelled with PKH26 according to manufacturer's instructions. Chemical inhibitors of internalization were used at 10 µM cytochalasin D, 30 µM PP1, or 50 µM LY294002, by incubation of RAW cells at 37°C for 30 minutes in RAW medium with serum immediately prior to the trogocytosis assay. 0.5% DMSO was used as a vehicle control. After adding RTX-Al488 coated Ramos cell suspensions to RAW cells, chemical inhibitors were restored to their appropriate concentrations. For PAO pretreatment of Ramos cells, cells were incubated with 30 µM PAO for 10 minutes, then washed twice to remove PAO before addition to RAW cells.

### Live microscopy of trogocytosis

RAW cells (1×10^6^ cells) were seeded on 25 mm diameter cover glass (Fisher Scientific), cultured overnight, then mounted in an Attofluor chamber (Molecular Probes) followed by imaging on a 37°C heated stage. RAW cell media was replaced with phenol red-free HEPES-buffered RPMI (HPMI) with serum (Wisent). Ramos cells (1×10^6^ cells) were labelled with PKH26 (in some experiments), coated with RTX-Al488, resuspended in HPMI and laid over RAW cells. Epifluorescent microscope images were acquired with a Hamamatsu ORCA camera attached to a Zeiss Axiovert 200M microscope, with 40×1.3NA oil and 100×1.4NA oil objectives. Alternatively, scanning confocal microscope images were acquired with a Zeiss LSM 510 with a 63×1.2NA water objective. Time lapse data was generated by imaging at 30s intervals. Care was taken to minimize exposure time for each field of view to reduce photobleaching and phototoxicity. Data were analyzed with AxioVision (Zeiss) and Volocity (Improvision) software packages. For observation of Ramos cell motility, cells were viewed either on cover glass on which RAW cells had been cultured, or on cover glass pre-incubated with RAW media (DMEM with 10% FBS), since Ramos cells adhered and spread irreversibly when plated on fresh cover glass in serum-free conditions. Ramos cell polarization was scored by observation of lamellipodial extensions by differential interference contrast microscopy.

### Flow cytometry-based trogocytosis assay

Ramos cells were labelled with the fluorescent lipid dye PKH26 (in some experiments), then opsonized with RTX-Al488. RAW cells were seeded in 6 well plates (10^6^ cells/well) and cultured overnight. RAW cells were treated with appropriate chemical inhibitors for 30 minutes at 37°C and then cooled to 4°C. RAW medium was then replaced with Ramos cell suspensions, supplementing inhibitors where necessary. Ramos cells (2–4×10^6^ cells per well) were provided to obtain a 2∶1 target: trogocyte ratio and were allowed to settle onto RAW cells for 10 minutes at 4°C. Trogocytosis was initiated by moving cells to a 37°C 5% CO_2_ incubator, and stopped by moving cell mixtures to 4°C. Remaining surface-bound rituximab on RAW cells was labelled with DL649-conjugated anti-human antibody (0.6 µg per 10^6^ cells) at 4°C for 10 minutes. Adherent cells were washed once with PBS. In some experiments, adherent cells were stained with fluorophore-conjugated CD11b at 4°C for 10 minutes and washed again. Adherent cells were then scraped, resuspended in PBS, and fixed with 2% paraformaldehyde prior to flow cytometry.

### Flow cytometry kinetic analysis

The RAW population in the detached adherent cells was analyzed for the acquisition and internalization of RTX. Cells were initially gated for the main population of cells by scatter, which identifies both RAW and residual Ramos cells. RAW cells were identified by CD11b positivity or by excluding Ramos cells as events high in both PKH26 and anti-human DL649 (Figure S1).

RAW cell events were analyzed by an external RTX (anti-human) versus total RTX plot. Quadrants were drawn according to the 0 minute condition to define a double negative (DN) population, and infer a double positive (DP) population and total RTX single positive (SP) population (See Figure 6A). These represent populations of RAW trogocytic intermediates and RAW cells that have trogocytosed and internalized RTX, respectively. Spillover events in the top left quadrant were not considered in the analysis. The exit of DN events into the DP population for conditions without chemical inhibitors was modelled by the exponential decay function 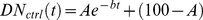
(1)


where *A* reflects the final proportion of RAW cells that respond (acquire RTX), and *b*, a rate constant. For chemical inhibition experiments (in which delay of transition to DP population was seen), the DN population was modelled by the sigmoidal function
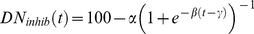
(2)


where *α* represents the proportion of responding RAW cells, *β*, the rate constant, and γ the time required for half of the responding RAW cells to respond. Coefficients were determined by least-squares analysis by Excel's Solver function. A two-tailed paired Student's t-test was used to determine significance of differences between chemically-inhibited and control conditions.

## Supporting Information

Figure S1 Gating strategies for distinguishing RAW and Ramos cells. RAW and Ramos cells were incubated for 10 min to allow conjugates to form, then adherent cells were washed, detached and processed for flow cytometry. (A) Intact RAW-Ramos conjugates did not persist after cell processing. While a population of RAW cells acquired anti-human staining, very few show staining equivalent in total fluorescence to Ramos cells, as would be expected for cell-cell conjugates (dotted box). (B) Cells were stained with anti-CD11b-PE to distinguish RAW cells from Ramos. (C) RTX and anti-human staining analysis with CD11b+ RAW cells shown in blue and CD11b- Ramos in red, showing distinct populations. (D) Alternative gating strategy used in experiments where Ramos cells were PKH-labelled. Ramos cells were identified based on high staining with anti-human and PKH (shaded gate). (E) RTX and anti-human staining analysis with RAW and Ramos gated as in (D) in blue and red, respectively, showing populations similar to those seen with CD11b staining.
(9.25 MB TIF)

Figure S2 Transfer of membrane is inhibited by cytochalasin D. PKH26 labelled, RTX-Al488 coated Ramos cells were coincubated with RAW cells for the times indicated. Co-transfer of RTX-Al488 and PKH26 occurs in the absence of cytochalasin D (top row). After treatment with cytochalasin D (bottom row), transfer is reduced, limited to RTX only, and observed only late in the reaction.
(4.32 MB TIF)

Video S1 Ramos morphology and RTX localization. Reversible changes in morphology coincide with the enrichment of RTX-Al488 (green) at the cell-substrate interface. Opposite the uropod, extensive membrane projections are observed. Scale bar 10 µm. Images acquired every 30s, played at 15fps (450× RT). Total time: 30 min.
(0.38 MB MP4)

Video S2 Ramos mobility. Live time lapse microscopy 90 minutes after the addition of RTX-Al488 (green) coated Ramos cells to RAW cells. A Ramos cell is shown moving along the glass surface while dynamically changing morphology. Asterisks indicate RAW cells performing trogocytosis of captured Ramos. Scale bar 10 µm. Images acquired every 30s, played at 15fps (450× RT). Total time: 30 min.
(2.29 MB MP4)

Video S3 Uropod capture and trogocytosis at the RAW-uropod interface. Movie of live microscopy experiment shown in Figure 2A. RTX-Al488 (green) labeled Ramos cells were incubated with RAW cells. Scale bar 10 µm. Images acquired every 30s, played at 15fps (450× RT). Total time: 29.5 min.
(1.24 MB MP4)

Video S4 Capture of Ramos cells and RTX-Al488 enrichment at interface. RTX-Al488 (green) labelled Ramos cells were incubated with RAW cells. Scale bar 10 µm. Images acquired every 30s, played at 15fps (450× RT). Total time: 30 min.
(1.51 MB MP4)

Video S5 Multiple Ramos captured by a single RAW cell. RAW cells (*) are able to trogocytose from many Ramos cells at once. RTX-depleted mobile end of Ramos cell was not captured by RAW (red arrow). Time lapse taken 30 min after the addition of RTX-Al488 coated Ramos cells to RAW cells. Scale bar 10 µm. Images acquired every 30s, played at 15fps (450× RT). Total time: 30 min.
(1.02 MB MP4)

Video S6 Streaming of trogocytosed RTX. Movement of trogocytosed RTX within RAW cells 1–1.5 hr after the addition of RTX-Al488 coated Ramos to RAW cells. Fading of fluorescence signal occurs as a result of photobleaching. Scale bar 10 µm. Images acquired every 30s, played at 15fps (450× RT). Total time: 30 min.
(2.57 MB MP4)

Video S7 RAW cells after trogocytosis. Live microscopy imaging 1 hr after addition of RTX-Al488 coated Ramos cells to RAW cells. RTX has collected in large vacuolar compartments in many cells. Scale bar 10 µm. Images acquired every 30s, played at 15fps (450× RT). Total time: 5 min.
(6.12 MB MP4)
