# Supplementary figures and images for: Dynamics of Macrophage Trogocytosis of Rituximab-Coated B Cells
Source: PLoS One. 2011 Jan 17;6(1):e14498. doi: 10.1371/journal.pone.0014498 (PMC3022012; doi:10.1371/journal.pone.0014498)

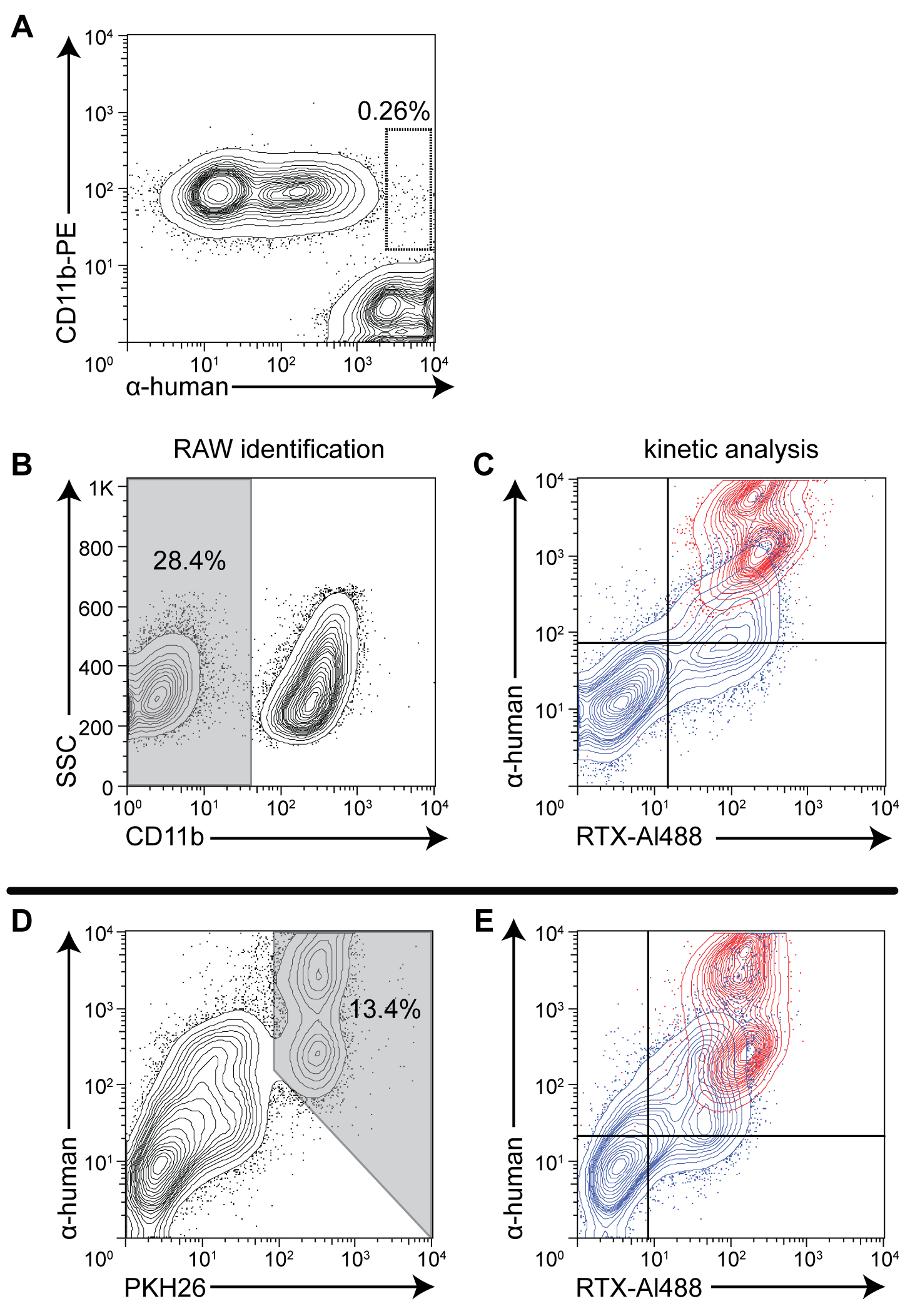

Supplement: Figure S1 — Gating strategies for distinguishing RAW and Ramos cells. RAW and Ramos cells were incubated for 10 min to allow conjugates to form, then adherent cells were washed, detached and processed for flow cytometry. (A) Intact RAW-Ramos conjugates did not persist after cell processing. While a population of RAW cells acquired anti-human staining, very few show staining equivalent in total fluorescence to Ramos cells, as would be expected for cell-cell conjugates (dotted box). (B) Cells were stained with anti-CD11b-PE to distinguish RAW cells from Ramos. (C) RTX and anti-human staining analysis with CD11b+ RAW cells shown in blue and CD11b- Ramos in red, showing distinct populations. (D) Alternative gating strategy used in experiments where Ramos cells were PKH-labelled. Ramos cells were identified based on high staining with anti-human and PKH (shaded gate). (E) RTX and anti-human staining analysis with RAW and Ramos gated as in (D) in blue and red, respectively, showing populations similar to those seen with CD11b staining. (9.25 MB TIF) [file pone.0014498.s001.tif]

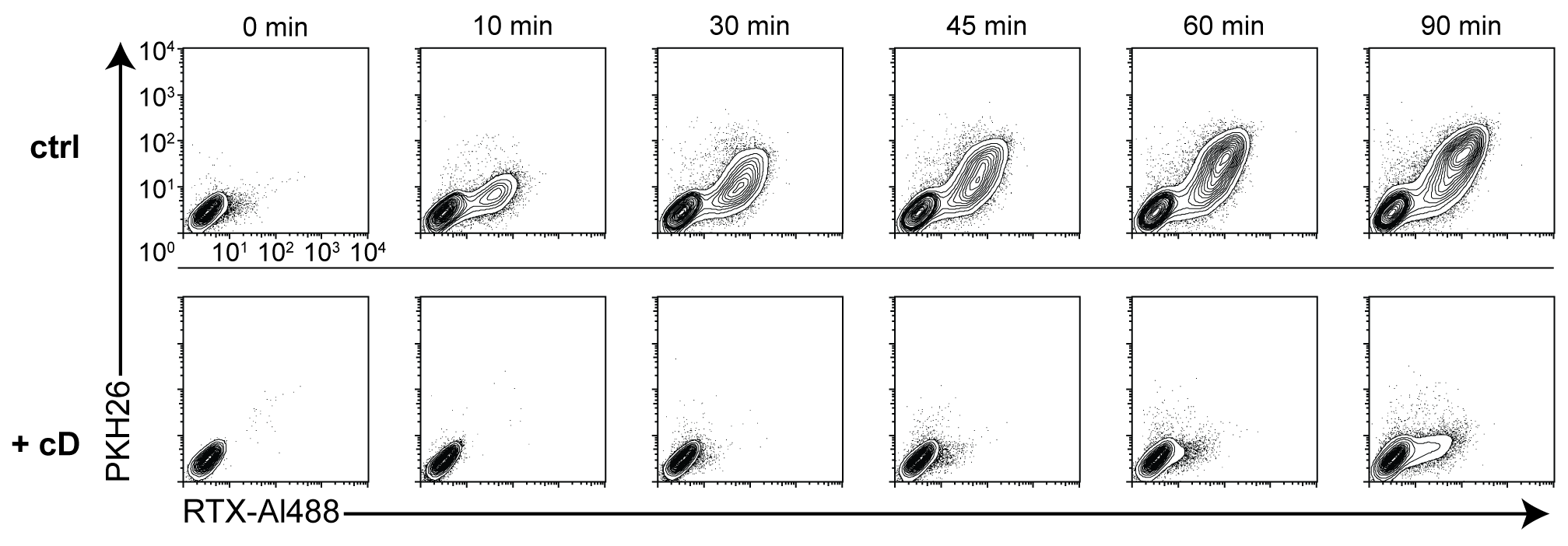

Supplement: Figure S2 — Transfer of membrane is inhibited by cytochalasin D. PKH26 labelled, RTX-Al488 coated Ramos cells were coincubated with RAW cells for the times indicated. Co-transfer of RTX-Al488 and PKH26 occurs in the absence of cytochalasin D (top row). After treatment with cytochalasin D (bottom row), transfer is reduced, limited to RTX only, and observed only late in the reaction. (4.32 MB TIF) [file pone.0014498.s002.tif]
